# Downregulation of Interleukin-13 Receptor α2 Inhibits Angiogenic Formation Mediated by Chitinase 3-Like 1 in Late Atherosclerotic Lesions of apoE^−/−^ Mice

**DOI:** 10.3389/fphys.2021.690109

**Published:** 2021-07-19

**Authors:** Qi Xue, Lei Chen, Jianwu Yu, Kewang Sun, Lifang Ye, Jianlei Zheng

**Affiliations:** ^1^Department of Cardiology, Zhejiang Provincial People's Hospital, People's Hospital of Hangzhou Medical College, Hangzhou, China; ^2^Department of Pathology, Zhejiang Provincial People's Hospital, People's Hospital of Hangzhou Medical College, Hangzhou, China; ^3^Key Laboratory of Endocrine Gland Diseases of Zhejiang Province, Zhejiang Provincial People's Hospital, People's Hospital of Hangzhou Medical College, Hangzhou, China

**Keywords:** angiogenesis, CHI3L1, IL-13Ra2, proliferation, signaling/signaling pathways

## Abstract

**Aim:** Chitinase 3-like 1 (CHI3L1) has the potential to prompt proliferation and angiogenic formation. Interleukin-13 receptor α2 (IL-13Rα2) was regarded as a receptor of CHI3L1; however, it is unknown whether CHI3L1 adjusts the neovascularization in late atherosclerotic lesions of apoE^**−/−**^ mice *via* IL-13Rα2.

**Methods:** Silicone collars were placed around one of the common carotid arteries of apoE^**−/−**^ mice fed with a high-fat diet. The mice were further injected with Ad.CHI3L1 alone or Ad.CHI3L1 + Ad.IL-13Rα2 shRNA through the caudal vein. The plaque areas in the whole aorta and aortic root were evaluated by Oil Red O staining and H&E staining. The contents of CD31, CD42b, and collagen in carotid plaques were investigated by immunohistochemistry and Masson trichrome staining. The role of CHI3L1 in migration and tube formation of human umbilical vein endothelial cells (HUVECs) was determined by transwell and Matrigel tests. The effect of CHI3L1 on the expression of AKT and extracellular signal-regulated kinase (ERK) was evaluated with the Western blot.

**Results:** The plaque loads in the aorta were significantly more extensive in apoE^**−/−**^ mice injected with Ad.CHI3L1 than those with Ad.CHI3L1 + Ad.IL-13Rα2 shRNA. CHI3L1 significantly increased the contents of CD31 and CD42b and decreased the element of collagen in late-stage atherosclerotic lesions of the carotid arteries. The effects of CHI3L1 on migration, tube formation, and upregulation of phospho-AKT and phospho-ERK of HUVECs were prohibited by inhibitors of phosphatidylinositol 3-kinase (PI3K) and mitogen-activated protein kinase kinase (MEK) as well as IL-13Rα2 shRNA.

**Conclusion:** To some extent, CHI3L1 promotes migration and tube formation of HUVECs and neovascularization in atherosclerotic plaques possibly mediated by IL-13Rα2 through AKT and ERK signal pathways.

## Introduction

Atherosclerosis is a complex and multifactorial pathology occurred in arterial walls. Endothelial dysfunction, foam cell formation, cholesterol deposition, inflammation, extracellular matrix synthesis, smooth muscle cell biological transition, and immature neovascularization of the plaque were involved in the initiation and development of atherosclerosis (Gimbrone and García-Cardeña, 2016; Wang and Khalil, 2018; Basatemur et al., 2019; Moroni et al., 2019). Recently, accumulating evidence showed that intraplaque angiogenesis played a pivotal role in plaque destabilization and plaque rupture (Badimon and Vilahur, 2014; Sedding et al., 2018). Erosion and rupture of atherosclerotic plaques are the main cause of acute cardiovascular effects, such as acute myocardial infarction, stroke, and sudden death (Bentzon et al., 2014; Hansson et al., 2015). Hence, exploring the underlying mechanism of intraplaque neovascularization is crucial for the prevention and treatment of cardiovascular diseases caused by atherosclerosis.

Chitinase 3-like 1 (CHI3L1), also named YKL-40, is a pro-inflammatory cytokine found in atherosclerotic lesions (Rathcke and Vestergaard, 2009). In clinical practices, it has been reported that elevated serum CHI3L1 was associated with acute coronary syndrome and cardiovascular morbidity and mortality (Nøjgaard et al., 2008; Rathcke et al., 2010; Ma et al., 2018). In our previous study, we demonstrated that overexpression of CHI3L1 increased inflammation and aortic plaque enlargement in early atherosclerotic lesions of apoE^**−/−**^ mice fed with a western diet (Chen et al., 2019). Except for the pro-inflammatory function, partial characterization of CHI3L1 is similar to growth factors such as insulin-like growth factor (IGF-1) and plays an important role in cell migration and proliferation (Recklies et al., 2002; Jacques et al., 2007). Noticeably, it has been demonstrated that IL-13Rα2 was one of the membrane receptors for CHI3L1 (He et al., 2013), and the function of CHI3L1 adjusting cell apoptosis, TGF-β1 production, and several signal pathways, including macrophage mitogen-activated protein kinase (MAPK), protein kinase B/AKT, and Wnt/β-catenin, was mediated by IL-13Rα2-dependent mechanisms (Lee et al., 2016; Abd El-Fattah et al., 2018; Zhou et al., 2018). It is well-known that the proliferation and migration of endothelial cells (ECs) are essential for angiogenesis in atherosclerotic plaques. Given the proangiogenic role of CHI3L1 in various tumor cells, it is necessary to understand whether CHI3L1 has a similar role in adjusting tube formation of human umbilical vein ECs (HUVECs) and neovascularization in late-stage atherosclerosis.

Hence, in order to verify this hypothesis, this *in vivo* and *in vitro* study explored the effects of CHI3L1 on migration and tube formation of HUVECs and on intraplaque angiogenesis. Furthermore, we explored the potential signal pathways of CHI3L1–IL-13Rα2 axis in adjusting neovascularization.

## Methods

### Animal Model

Twenty-four male apoE^**−/−**^ mice aged 8 weeks on the C57BL/6J background were purchased from Model Animal Research Center of Nanjing University (Nanjing, China). Mice were given access to the chow diet and water *ad libitum* for 2 weeks. At the age of 10 weeks, mice were randomly assigned into three groups (i.e., Ad.CHI3L1 group, Ad.CHI3L1 + Ad.IL-13Rα2 shRNA group, and control, *n* = 8, each group), and a silicone collar (Silastic Cat. No. 508-002, Dow Corning Corporation, USA) was placed around the left common carotid arteries (Zhi et al., 2013). Specifically, mice were anesthetized with pentobarbital, and the left common carotid arteries were isolated from the surrounding connective tissue. Collars were placed carefully around the left carotid arteries and tied with surgical silk thread, and the operation wound was sutured. After the successful construction of the carotid model, all apoE^**−/−**^ mice were provided with a high-fat diet (HD012; purchased from BiotechHD Co., Ltd., Beijing, China) for the subsequent 18 weeks. High-fat diet intake was closely monitored and did not differ among groups. At the age of 18 and 22 weeks, each group was injected into 200 μl of different reagents per time [Ad.CHI3L1 and Ad.CHI3L1 + Ad.IL-13Rα2 shRNA provided by Shandong ViGene Biosciences (Jinan, Shandong, China), the corresponding virus titer was 1.0 × 10^10^/ml], and the control group was injected into 200 μl of phosphate-buffered saline (PBS) through tail veins. The sequence of IL-13Rα2 shRNA for mice is as follows: GCCTCCATGTGGATCTGAAC ATTCAAGAGATGTTCAGATCCACATGGAGGCTTTTTT. Mice were sacrificed at 28 weeks of age, and the method of euthanasia was carried out through intraperitoneal injection of sodium pentobarbital overdose. Aorta and carotid arteries were collected and fixed in paraformaldehyde and embedded in paraffin for atherosclerosis analysis. This study was approved by the Ethics Committee of the Animal Research Institute of Zhejiang Provincial People's Hospital (Zhejiang, China). All the procedures were performed in accordance with the guidelines of the National Institutes of Health Guide for the Care and Use of Laboratory Animals.

### Analysis of Atherosclerotic Lesions

Adventitial fatty tissues were carefully removed from the aorta using pincettes. Aortas were incised longitudinally from the ascending aorta to the abdominal aorta and fixed in 4% paraformaldehyde for 36 h. Later, the fixed aortas were successfully stained with Oil Red O (Sigma-Aldrich, USA). Then, the proportion of atherosclerotic plaques to the total aortic areas was measured according to Oil Red O staining. Cross-sections of the aortic root and carotid arteries with silicon collar placement were stained with H&E (Maiwei, Xiamen, China) to evaluate the size of atherosclerotic lesions. Collagen area in carotid plaques was assessed with Masson trichrome staining. Quantitative analysis of lesions was performed with Image-Pro Plus 6.0 software.

### Immunohistochemistry Analysis for Neovascularization in Carotid Arteries

Paraformaldehyde-fixed, paraffin-embedded left common carotid arteries with silicon collar placement were serially cut into 10 μm thick sections for the morphometric analyses. Histological sections from the left common carotid arteries were treated with 3% hydrogen peroxide to block endogenous peroxidase activity, and immunohistochemical staining was performed with CD31 and CD42b (both are of 1:100 dilution, ab28364, Abcam, Cambridge, UK; 12860-1-AP, Proteintech, USA, respectively), then incubated with a biotinylated secondary antibody, and finally counterstained with Mayer's H&E. All cross-sections were analyzed under an upright microscope (Nikon, Tokyo, Japan). The positive areas were measured in three non-overlapping fields and analyzed with Image-Pro Plus 6.0 software.

### Cell Culture

Human umbilical vein ECs were purchased from the Shanghai Institutes for Biological Sciences, the Chinese Academy of Sciences (Shanghai, China). HUVECs were cultured in DMEM/F12 (Gibco, USA) with 10% fetal bovine serum (FBS) (Thermo-Fisher Scientific, Waltham, USA) supplemented with endothelial growth factor (ScienCell Research Laboratories, Carlsbad, CA, USA), 100 U/ml of penicillin, and 100 μg/ml of streptomycin at 37°C with 5% of CO_2_. HUVECs in this study were divided into control (HUVECs without additional treatment), CHI3L1 (400 ng/ml of recombinant CHI3L1, ab182706, Abcam, Cambridge, UK) group, mitogen-activated protein kinase kinase (10 μM of MEK, PD98059, MCE, USA) inhibitor group, phosphoinositide 3-kinase (10 μM of PI3K, LY294002, MCE, USA) inhibitor group, negative control (NC)-shRNA group, Ad.IL-13Rα2 shRNA group, NC shRNA + CHI3L1 group, and Ad.IL-13Rα2 shRNA + CHI3L1 group. The sequence of IL-13Rα2 shRNA for *Homo sapiens* is as follows: GATCCGGTATTGACTCAACAGTTTCCTTCAAGAGAGGAAACTGTTGAGTCAATACCTTTTTTA.

### Cell Counting Kit-8 Assay and Interference Effect of Ad.IL-13Rα2 shRNA

The proliferation of HUVECs regulated by different concentrations of CHI3L1 at different time points (24 and 48 h) was determined using Cell Counting Kit-8 (CCK-8) (Beyotime, Nanjing, Jiangsu, China). Of note, 2 × 10^3^ cells were seeded into 96-well plates. Then, 20 μl solutions of CCK-8 reagent were added to each well at 24 and 48 h. The cells were cultured with the CCK-8 assay for 1 h at 37°C. Finally, the absorbance value of HUVECs was measured with a microplate reader at 450 nm. In addition, HUVECs transfected with Ad.IL-13Rα2 shRNA or negative control Ad.shRNA (NC-shRNA) grown in 60-mm dishes were cultured for 48 h. Then, the cells were harvested, and the inhibition effect of Ad.IL-13Rα2 shRNA on membrane protein of IL-13Rα2 (1:1,000 dilution, 11059-1-AP, Proteintech, USA) was measured with Western blotting. In addition, the expression of IL-13Rα2 in the aorta was analyzed by the immunohistochemistry assay (1:200 dilution, 11059-1-AP, Proteintech, USA).

### Transwell Assay

The migration of HUVECs was evaluated with the transwell system (Corning Costar, MA, USA). Of note, 1 × 10^5^ HUVECs at 95% confluence were seeded in the upper chambers for 12-h attachment. Cells were subjected to MEK inhibitor for 1 h, PI3K inhibitor for 1 h, and corresponding shRNAs for 48 h and were then added to recombinant protein CHI3L1 culture for another 24 h. After the cells were dealt with the corresponding procedure mentioned above, the medium in upper chambers was switched to a medium with 0.5% of FBS and that in the lower chamber was switched to a medium with 1% of FBS. Following 12 h of incubation, the cells on the bottom were fixed with 4% paraformaldehyde and stained with 0.1% crystal violet, and the unattached cells were removed from the upper surface by gentle scrubbing. After three washes, the migrated cells were obtained using an Olympus microscope (magnification 200×) equipped with Leica Application Suite Program (Leica Microsystems, Switzerland). Each treatment was repeated three times.

### Tube Formation Assay

A tube formation assay was performed to evaluate the effect of CHI3L1 on the angiogenesis ability of HUVECs. In brief, the cells were cultured in a medium containing 10% of FCS pre-cultured with MEK inhibitor, PI3K inhibitor for 1 h, and corresponding shRNAs for 48 h, followed with or without 400 ng/ml of CHI3L1 for 24 h. Finally, the cells seeded into a 96-well plate at a density of 5 × 10^3^ cells/well were overlaid with Matrigel substrate (BD Biosciences, CA), and were incubated at 37°C for 6 h. Capillary tube structures were observed and quantified (Hou et al., 2017). Representative images were captured using Leica Application Suite Program (magnification ×100). The value of tubular branches representing the capacity of angiogenesis was determined using Image J software (National Institutes of Health, Bethesda, MD, USA).

### Protein Expression of AKT and Extracellular Signal-Regulated Kinase

The procedures for the HUVEC experiment are the same as transwell and tube formation assays. Processed cells were lysed by using the radioimmunoprecipitation assay lysis buffer (Waltham, MA, USA) at 4°C for 30 min and subjected to 12,000 rpm centrifugation at 4°C for 5 min. The protein concentration was measured by using BCA Protein Assay Kit (Waltham, MA, USA). The lysates were separated by 10% sodium dodecyl sulfate–polyacrylamide gel electrophoresis (SDS-PAGE), transferred to polyvinylidene fluoride (PVDF) membranes, blocked with 5% skimmed milk for 1 h at room temperature, washed, and incubated overnight with primary antibodies at recommended dilutions. The antibodies were used in this study including anti-p- ERK1/2, anti-ERK1/2 (1:2,000, Cat No: 5683T, CST, USA; 1:1,000, Cat No: ab65142, Abcam, Cambridge, UK, respectively), anti-AKT, anti-pAKT (1:300, Cat No: 4691T, CST, USA; 1:100, Cat No: 4060T, CST, USA, respectively), and anti-glyceraldehyde-3-phosphate dehydrogenase (anti-GAPDH) (1:10,000, Cat No: ab181602, Abcam, Cambridge, UK). After washing, blots were probed with horseradish peroxidase-conjugated secondary antibody (Abcam) for 1 h. Finally, the content of proteins was measured by an enhanced chemiluminescence system obtained from Pierce Biotechnology (Waltham, USA).

### Statistical Analysis

The data are presented as means ± SD. A two-tailed Student's *t*-test was used to determine significance. Differences were considered significant for *p* < 0.05. Analyses were done using the statistical SPSS 11.0 software (SPSS, Inc., Chicago, IL, USA).

## Results

### The Plaque Areas of Atherosclerotic Lesions in Different Groups

**Figure 1:**
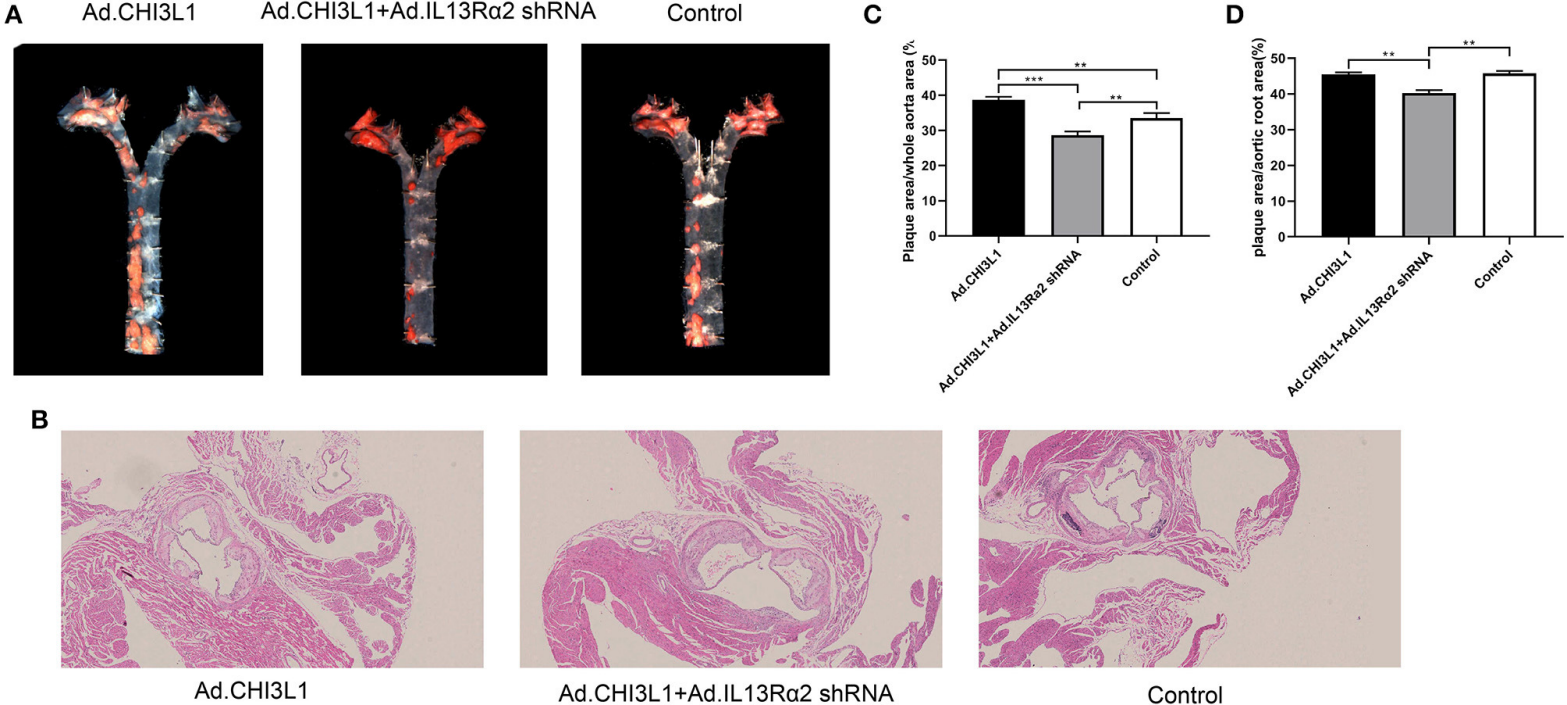
The effect of Chitinase 3-like 1 (CHI3L1) and Interleukin-13 Receptor α2 (IL-13Rα2) shRNA on the atherosclerotic load of aortas from apoE^**−/−**^ mice fed with a high-fat diet. General Oil red O-stained aortas and ratio of plaque areas to the whole aorta among three groups **(A,C)**. H&E stained sections of the aortic root and quantification of cross-sectional areas of plaque in the aortic roots **(B,D)**. Data were presented as mean ± SD (*n* = 3 per group). **p < 0.01 and ***p < 0.001.

**Figure 2:**
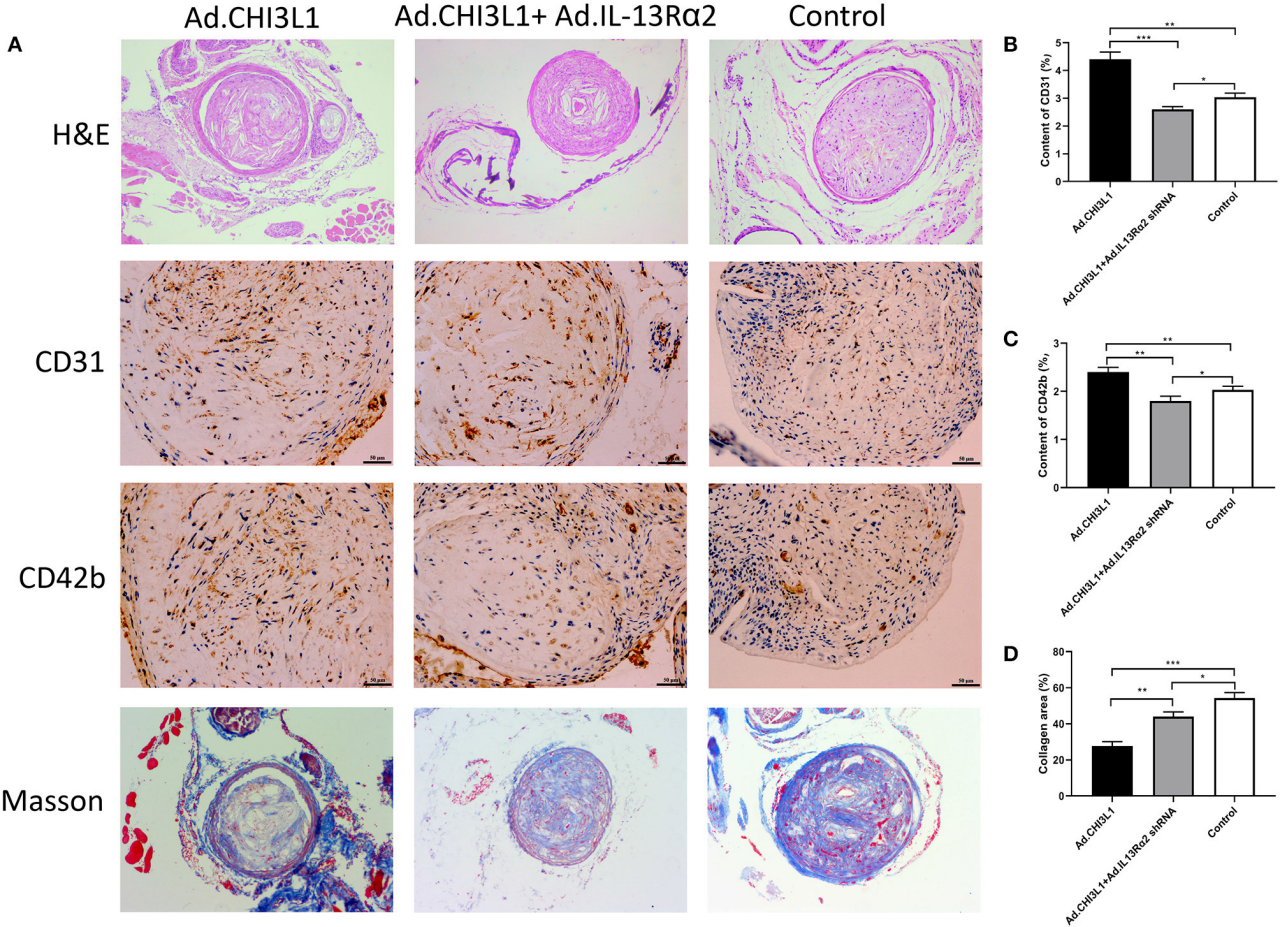
Atherosclerotic lesion and expression of CD31, CD42b, and collagen in carotid plaques of apoE^**−/−**^ mice pretreated with carotid collar placement. Representative H&E staining, immunohistochemical images of CD31 and CD42b, and Masson trichrome staining in carotid plaques **(A)**. Quantification of CD31, CD42b, and collagen in atherosclerotic lesions **(B–D)**. Data were presented as mean ± SD (*n* = 3 per group). *p < 0.05, **p < 0.01, and ***p < 0.001.

To investigate the effect of CHI3L1 on atherosclerosis development mediated by IL-13Rα2, apoE^**−/−**^ mice aged 8 weeks were fed with a chow diet of 2 weeks and then were followed with a western diet for 18 weeks. The results from Oil Red O staining which displayed the atherosclerotic plaques in the entire aorta from the group of overexpression of CHI3L1 were significantly larger than those in controls and Ad.CHI3L1 + IL-13Rα2 shRNA group (38.7 ± 0.85% vs. 33.5 ± 1.40% and 28.7 ± 0.98%, *p* = 0.005 and *p* < 0.001, respectively) (Figures 1A,C). The results of H&E staining showed that the area of atherosclerotic plaques in cross-sections of the aortic root was more extensive in Ad.CHI3L1 group than that in Ad.CHI3L1 + IL-13Rα2 shRNA group (45.5 ± 0.51% vs. 40.3 ± 0.79%, *p* = 0.001), although there was no statistically significant difference between Ad.CHI3L1 group and control (45.5 ± 0.51% vs. 45.7 ± 0.70%, *p* > 0.05) (Figures 1B,D). We speculated that the prolonged Western diet offsets the effect of the overexpression of CHI3L1 on atherosclerotic enlargement. Due to prolonged western diet and silicone collar placement, the whole lumen of carotid arteries was nearly completely filled with plaques among three groups (Figure 2A). However, compared with the controls, the collagen content in atherosclerotic lesions of carotid arteries significantly decreased in mice with the overexpression of CHI3L1 (27.7 ± 2.52% vs. 54.3 ± 3.05%, *p* < 0.001) (Figures 2A,D).

### The Expression of CD31 and CD42b in Carotid Plaques

Due to the proangiogenic role of CHI3L1 in various tumor cells, we determined to explore whether CHI3L1 also mediates neovascularization in atherosclerotic plaques. The representative images of the expression of CD31 and CD42b in carotid plaques are shown in Figure 2A. Intraplaque CD31 representing microvessel numbers was significantly upregulated in apoE^**−/−**^ mice injected with Ad.CHI3L1 compared with those injected with Ad.CHI3L1 + Ad.IL-13Rα2 shRNA and controls (4.40 ± 0.26% vs. 2.60 ± 0.10% and 3.03 ± 0.15%, *p* < 0.001 and *p* = 0.001, respectively) (Figure 2B). Since the newly formed vessels in atherosclerotic plaques were immature, they are liable to be leaky and induce intraplaque hemorrhage. As expected, the distribution of CD42b in intraplaque was remarkably increased in apoE^**−/−**^ mice with Ad.CHI3L1 than that in control group (2.40 ± 0.10% vs. 2.03 ± 0.08%, *p* = 0.007). However, compared with Ad.CHI3L1, the expression of CD42b mice with Ad.CHI3L1 + Ad.IL-13Rα2 shRNA was significantly decreased (2.40 ± 0.10% vs. 1.80 ± 0.10%, *p* = 0.002) (Figure 2C).

### The Role of CHI3L1 in Cell Proliferation and Interference Effect of Ad.IL-13Rα2 shRNA

**Figure 3:**
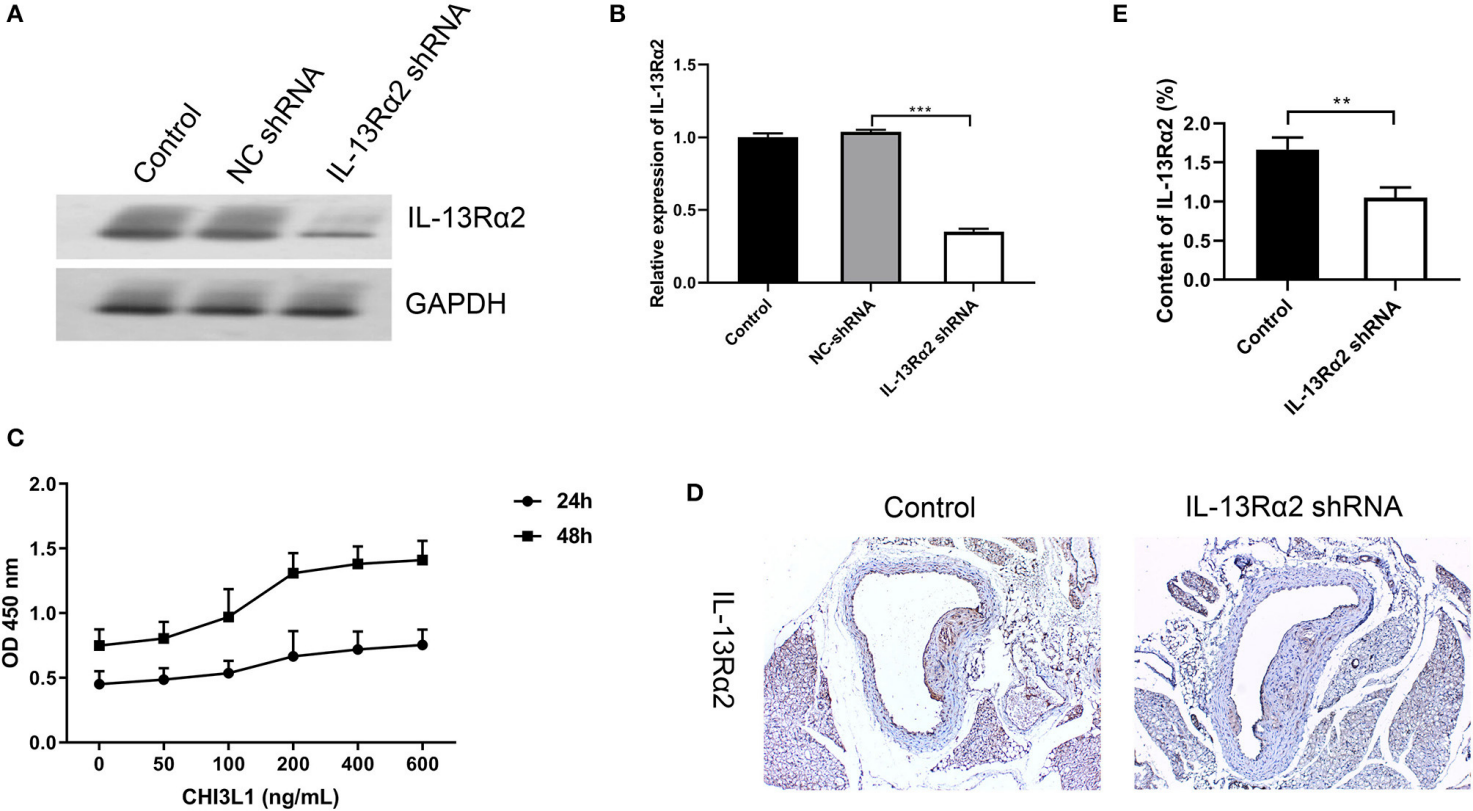
Interference effect of Ad.IL-13Rα2 shRNA and optimal concentration of CHI3L1 for proliferation. Interference effect of Ad.IL-13Rα2 shRNA on membrane protein of IL-13Rα2 in human umbilical vein endothelial cells (HUVECs) measured by Western blotting **(A,B)**. The CCK-8 assay was performed to investigate the proliferation of HUVECs with different concentrations of CHI3L1 at 24 and 48 h, respectively **(C)**. The inhibition effect of Ad.IL-13Rα2 shRNA on the expression of IL-13Rα2 in the aorta was analyzed by immunohistochemistry **(D,E)**. Data were presented as mean ± SD (*n* = 3 per group). **p < 0.01 and ***p < 0.001.

We evaluated the proliferation effects of CHI3L1 on HUVECs with different concentration gradients; as a result, the role of CHI3L1 in proliferation was dependent on concentration and time. The ideal concentration for CHI3L1 to increase HUVECs proliferation was 400 ng/ml in this study (Figure 3C), and this concentration was selected for subsequent cell experiments. In contrast, we measured the knockdown effect of Ad.IL-13Rα2 shRNA on IL-13Rα2 in HUVECs. The result showed that the membrane protein expression of IL-13Rα2 was significantly inhibited by Ad.IL-13Rα2 shRNA (1.00 ± 0.03 vs. 0.35 ± 0.02, *p* < 0.001) (Figures 3A,B). Furthermore, we found that the expression of IL-13Rα2 in aorta in mice injected with Ad.IL-13Rα2 shRNA was lower than that in controls (1.05 ± 0.13 vs. 1.67 ± 0.15, *p* = 0.006) (Figures 3D,E).

### The Effect of Downregulation of IL-13Rα2 on the Migration of HUVECs Treated With Recombinant Protein CHI3L1

**Figure 4:**
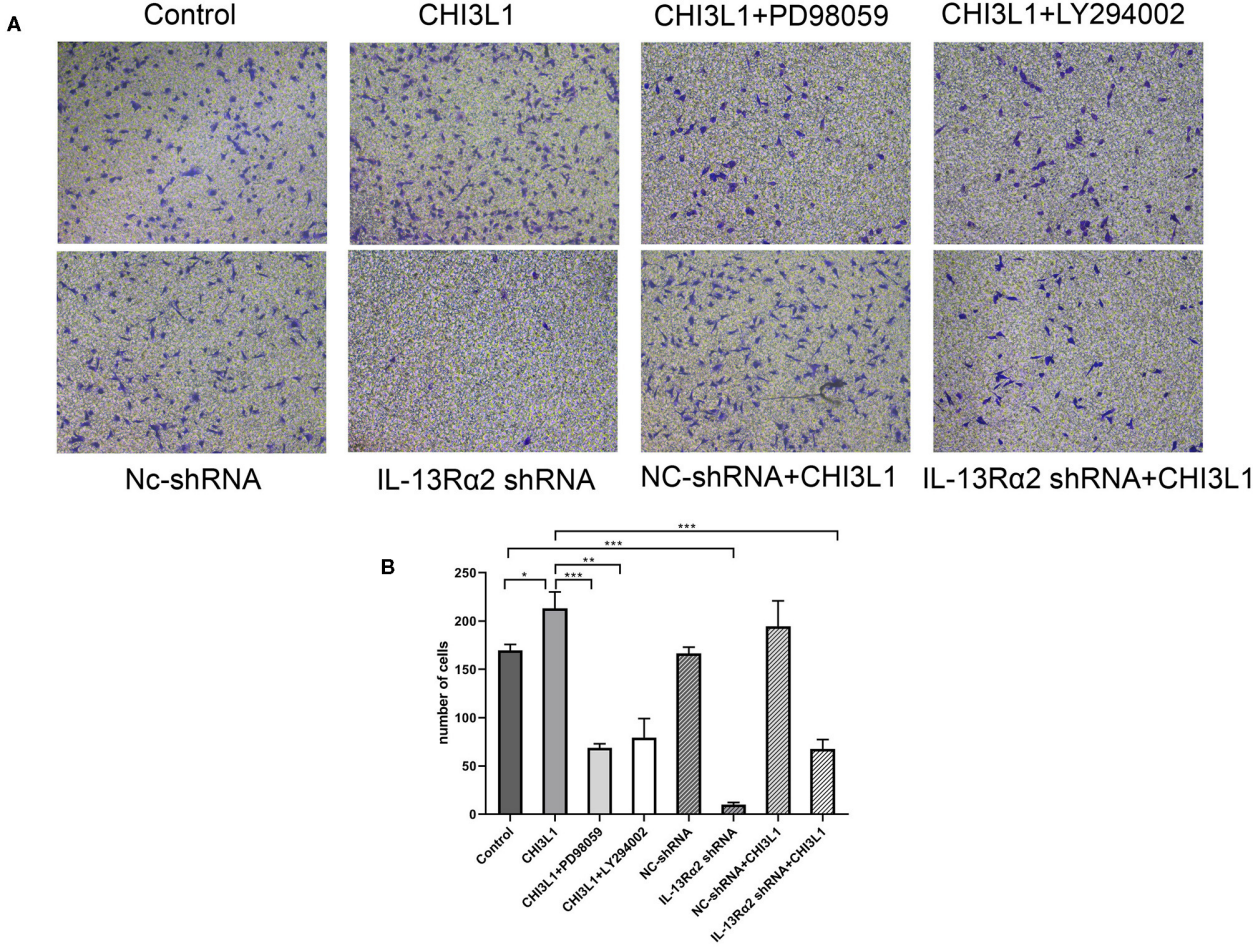
The migration of HUVECs was measured *via* transwell assay (magnification 200×). The effects of CHI3L1 on HUVECs migration were tested in the presence or absence of IL-13Rα2 shRNA, MEK (PD98059) inhibitor, and PI3K (LY294002) inhibitor **(A)**. The number of migration cells was calculated **(B)**. Data were presented as mean ± SD (*n* = 3 per group). *p < 0.05, **p < 0.01, and ***p < 0.001.

To assess the effects of CHI3L1 on the migration of HUVECs, transwell and Matrigel assays were utilized. The HUVECs were pre-cultured with recombinant protein of CHI3L1 remarkably prompt migration of ECs (213.00 ± 17.00 vs. 169.67 ± 6.03, *p* = 0.014) (Figures 4A,B). However, the migration of HUVECs was significantly decreased after pretreatment with Ad.IL-13Rα2 shRNA (169.67 ± 6.03 vs. 9.67 ± 2.52, *p* < 0.001) (Figures 4A,B). In addition, considering the signaling pathways of AKT and ERK playing an important role in adjusting cell migration and proliferation, HUVECs were pretreated with inhibitors of MEK (PD98059) and PI3K (LY294002) before CHI3L1 stimulation. As a result, similar to Ad.IL-13Rα2 shRNA, these two inhibitors showed notable inhibition effect on the migration of HUVECs (213.00 ± 17.00 vs. 68.67 ± 4.16 and 79.33 ± 19.76, *p* < 0.001 and *p* = 0.001, respectively) (Figures 4A,B).

### The Effect of Downregulation of IL-13Rα2 on the Tubular Formation of HUVECs Treated With Recombinant Protein CHI3L1

**Figure 5:**
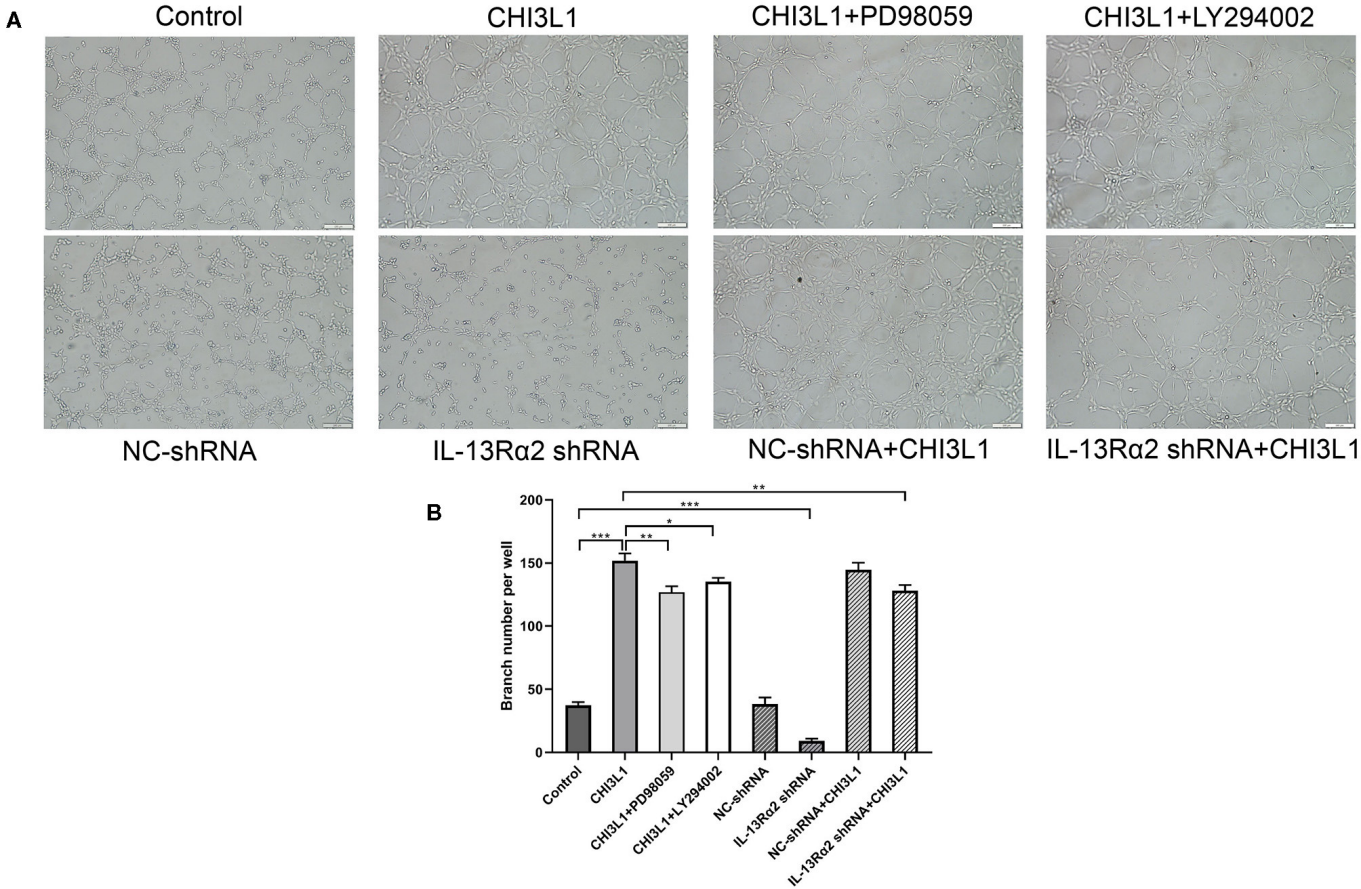
CHI3L1 prompted angiogenesis of HUVECs *in vitro* (magnification 100×). Representative images of angiogenesis of HUVECs. IL-13Rα2 shRNA, MEK (PD98059) inhibitor, and PI3K (LY294002) inhibitor inhibited the angiogenesis capacity stimulated with CHI3L1 **(A)**. The qualification of branch points number was calculated **(B)**. Data were presented as mean ± SD (*n* = 3 per group). *p < 0.05, **p < 0.01, and ***p < 0.001.

Generally, vascular endothelial proliferation and tubular formation are the initial processes of angiogenesis. We further investigated the proangiogenic response of CHI3L1 by examining the formation of tube-like capillary structures of HUVECs in matrix-gel using the recombinant protein of CHI3L1 with a concentration of 400 ng/ml. As expected, recombinant protein CHI3L1 increased the tubular formation of HUVECs, in comparison with control (152.00 ± 5.57 vs. 37.33 ± 2.52, *p* < 0.001) (Figures 5A,B). However, the ability of capillary tube formation was remarkably inhibited in HUVECs transfected with Ad.IL-13Rα2 shRNA (37.33 ± 2.52 vs. 9.00 ± 2.00, *p* < 0.001) (Figures 5A,B). Similarly, inhibitors of MEK (PD98059) and PI3K (LY294002) also decreased the ability of angiogenesis of HUVECs mediated by the recombinant protein of CHI3L1 (152.00 ± 5.57 vs. 127.00 ± 4.58 and 135.33 ± 3.06, *p* = 0.004 and *p* = 0.01, respectively) (Figures 5A,B).

### The Effect of IL-13Rα2 on Phosphorylation of ERK and AKT Protein Expression of HUVECs Treated With Recombinant Protein CHI3L1

**Figure 6:**
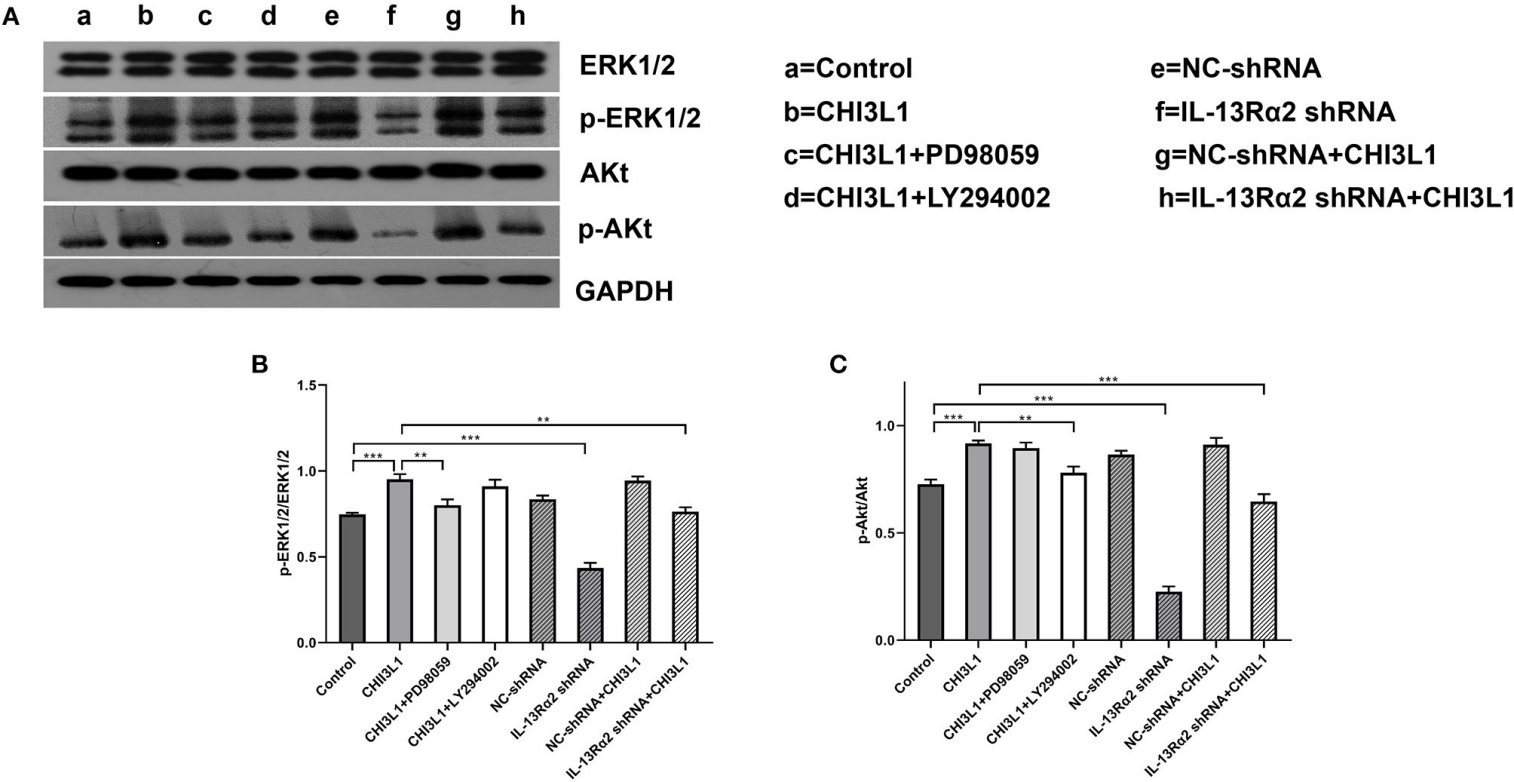
Western blot analysis of ERK and AKT in HUVECs. CHI3L1 prompted the phospho-ERK and phospho-AKT expression of HUVECs, and IL-13Rα2 shRNA decreased expression of phospho-ERK and phospho-AKT **(A)**. The expression ratio of phosphorylated ERK and AKT to total ERK and AKT was determined by densitometric analysis **(B,C)**. Data were presented as mean ± SD (*n* = 3 per group), **p < 0.01 and ***p < 0.001.

The Western blot analysis is shown in Figure 6A. The gray scale data proved that the levels of pERK/ERK (0.95 ± 0.03 vs. 0.75 ± 0.01, *p* < 0.001) and pAKT/AKT (0.92 ± 0.01 vs. 0.73 ± 0.02, *p* < 0.001) between CHI3L1 group and control group at 24 h were statistically significant (Figures 6B,C). However, IL-13Rα2 shRNA inhibited the expression of pERK/ERK (0.95 ± 0.03 vs. 0.76 ± 0.02, *p* = 0.001) and pAKT/AKT (0.92 ± 0.01 vs. 0.65 ± 0.03, *p* < 0.001) mediated by CHI3L1 (Figures 6B,C). As expected, the effects of inhibitors of ERK and AKT on restraining corresponding phosphorylation expression of ERK (0.95 ± 0.03 vs. 0.80 ± 0.03, *p* = 0.004) and AKT (0.92 ± 0.01 vs. 0.78 ± 0.03, *p* = 0.002) were remarkable (Figures 6B,C). These results suggested that CHI3L1 enhances neovascularization in late atherosclerotic lesions of apoE^**−/−**^ mice mediated, at least in part, by cell membrane receptor of IL-13Rα2 through ERK and AKT signaling pathways.

## Discussion

In this study, we investigated the role of CHI3L1 in regulating migration and angiogenesis of HUVECs and prompting atherosclerotic neovascularization in carotid plaques of apoE^**−/−**^ mice fed with a high-fat diet and pretreated with carotid collar placement. As a result, we observed that HUVECs stimulated with CHI3L1 enhanced EC migration and angiogenesis through IL-13Rα2 dependent pathways and its downstream signaling molecules of AKT and ERK were activated. In *in vivo* studies, neovascularization in carotid plaques was highly increased, mediated by the overexpression of CHI3L1, and the content of atherosclerotic plaques in aorta from mice with the overexpression of CHI3L1 was significantly higher than that from mice with knockdown of IL-13Rα2. However, overexpression of CHI3L1 significantly decreased the collagen content in plaques of carotid arteries.

Recently, CHI3L1 was regarded as a pro-inflammatory and proliferation cytokine and was associated with the initiation and progress of atherosclerosis (Rathcke and Vestergaard, 2009; Zheng et al., 2010). In clinical practices, studies demonstrated that elevated serum CHI3L1 levels were independently associated with coronary artery disease, and patients with the severity of coronary atherosclerosis had higher CHI3L1 levels (Kucur et al., 2007; Sui and Gao, 2013). Moreover, the increased concentration of serum CHI3L1 was closely associated with all-cause and cardiovascular mortality in an elderly population (Rathcke et al., 2010). All the above studies showed that CHI3L1 was probably a biomarker for atherosclerosis and unstable plaques. Recently, we demonstrated that the overexpression of CHI3L1 exaggerated the inflammatory reaction in early atherosclerotic lesions of apoE^**−/−**^ mice fed with a high-fat diet, and the serum CHI3L1 level was higher in apoE^**−/−**^ mice with an injection of Ad.CHI3L1 than that in controls (Chen et al., 2019). Another study showed that increased CHI3L1 expression exacerbated atherosclerosis by mediating EC inflammation and vascular smooth muscle cell (VSMC) activation (Jung et al., 2018). Except for the role of pro-inflammation, it was reported that CHI3L1 modulated vascular EC morphology by promoting the formation of branching tubules (Malinda et al., 1999). In fact, the role of CHI3L1 in eliciting angiogenesis has been extensively demonstrated in various tumor cells (Libreros et al., 2013; Ngernyuang et al., 2018). However, whether CHI3L1 prompts the angiogenesis of late atherosclerosis in animal models, as well as the concrete mechanism for CHI3L1 involved in atherosclerosis, is still unknown.

Neovascularization in atherosclerotic plaques means the unstable state, since the newly formed microvessels are often presented with immaturity and high permeability, permitting inflammatory cytokines to infiltrate the atherosclerotic plaques and induce hemorrhage (Jeney et al., 2014; Xu et al., 2015). In contrast, the inflammatory state is a strong inducer of angiogenesis as it promotes the release of inflammatory cytokines that facilitate the initiation and development of angiogenesis (Khurana et al., 2005). Incomplete endothelium structure leads to increased vascular permeability, which is inseparable for chronic inflammation and tumor angiogenesis. In this study, we demonstrated that CHI3L1 enhanced neovascularization in carotid plaques and migration and tubular formation of HUVECs, which reflected that CHI3L1 had a role in adjusting angiogenesis in atherosclerosis. Consistent with the previous study (van Lammeren et al., 2012), we found that CD42b, as a hemorrhage biomarker, was correspondingly increased with the number of neovascularization in atherosclerotic plaques. In addition, the plaque load in aortic arteries was higher in mice with recombinant adenovirus expressing CHI3L1 than that in mice with PBS. In fact, the contents of atherosclerotic plaque in the cross-sections of aortic root were similar between controls and the group of CHI3L1 overexpression. We speculated that this phenomenon may be associated with a prolonged western diet, which offsets the effect of CHI3L1 overexpression on atherosclerotic progression. The increased angiogenesis and hemorrhage in atherosclerotic plaques mean that the unstable plaques and are liable to adverse cardiovascular effects.

In recent years, IL-13Rα2 was considered as a membrane receptor for CHI3L1, and it was reported that the function of CHI3L1 adjusted proliferation and TGF-β1 production through IL-13Rα2-dependent mechanisms (He et al., 2013; Lee et al., 2016). In this study, we explored whether CHI3L1 plays a crucial role in the angiogenesis of HUVECs and neovascularization in carotid plaques through IL-13Rα2. As expected, we found that the knockdown of IL-13Rα2 significantly reduced the effects of CHI3L1 on the migration and tube formation of HUVECs. In this *in vivo* study, the present results indicated that mice injected with Ad.IL-13Rα2 shRNA showed remarkably decreased angiogenesis and hemorrhage in atherosclerotic plaques.

The signaling pathways of ERK and AKT were the main molecular adjusting networks in promoting the proliferation of HUVECs (Secchiero et al., 2003; Song et al., 2018). The effects of CHI3L1 on angiogenesis through focal adhesion kinase (FAK)/AKT have been mentioned in colonic epithelial cells (Chen et al., 2011). Another study indicated that the signaling pathway of MAPK also plays a critical role in angiogenesis stimulated by CHI3L1 in the bronchial epithelium (Tang et al., 2013). In this study, we further explored the potential molecular mechanism between CHI3L1 and its receptor of IL-13Rα2 in HUVCEs. As expected, the knockdown of IL-13Rα2 or inhibitors of ERK and AKT notably reduced the migration and angiogenesis of HUVECs and inhibited the expression of pAKT and pERK mediated by CHI3L1.

## Conclusions

This study indicated that the effects of CHI3L1 on migration and tube formation of HUVECs were partly regulated by IL-13Rα2 through ERK and AKT signal pathways. In contrast, according to our findings, we speculated that CHI3L1 as a prognostic factor for cardiovascular diseases may be associated with its role in promoting angiogenesis and forming unstable atherosclerotic plaques. In order to more powerfully explain the hypothesis, constructing animal experiments through gene knockout of CHI3L1 and/or IL-13Rα2 to clarify this mechanism is indispensable in the following studies. Designing antagonizing peptides, screening for potential small molecule agents, and developing monoclonal antibodies to inhibit CHI3L1 need to be considered.

However, there are several major limitations in this study. Although neovascularization in atherosclerotic lesions was regarded as a factor for unstable plaques, anti-angiogenic drugs caused different effects in animal experiments and clinical trials. Hence, the role of angiogenesis in plaques and the therapy for atherosclerotic angiogenesis need to be investigated (Camaré et al., 2017). In this study, we did not conclude the association between cardiovascular effects and neovascularization because no mice suffered from sudden death. In fact, it is reported that most of the plaques may be silent throughout life but only a few lesions may provoke thrombotic risk and lead to life-threatening complications (Hansson et al., 2015). Doubts still remain whether the various findings of animal experiments will cause similar cardiovascular prognosis in human given the key differences in lipoprotein metabolism and inflammation (Bentzon and Falk, 2010). Accordingly, whether the effects of CHI3L1 on angiogenesis of animal models mediated by IL-13Rα2 are similar to human beings needs to be further verified. In addition, the expression of CHI3L1 and IL-13Rα2 shRNA mediated by adenovirus are intermittent and unstable. Furthermore, adenovirus was administrated after 8 weeks of western diet, which only reflects the proangiogenic effect of CHI3L1 on formed atherosclerotic plaques. Hence, mice with knockout of CHI3L1 and/or IL-13Rα2 will more comprehensively illuminate the role of CHI3L1 in the initiation and progression of atherosclerosis and neovascularization in atherosclerotic plaques through the IL-13Rα2 pathway.

## Data Availability Statement

The raw data supporting the conclusions of this article will be made available by the authors, without undue reservation.

## Ethics Statement

The present study was approved by the Ethics Committee of the Animal Research Institute of ZheJiang Provincial People's Hospital.

## Author Contributions

JZ and QX designed the study. The immunohistochemistry analysis was performed by LC. The animal model was carried out by KS and QX. Cell experiments were done by LY. JY wrote the first draft of the manuscript. Data analysis was performed by JZ. All authors read and approved the final manuscript.

## Conflict of Interest

The authors declare that the research was conducted in the absence of any commercial or financial relationships that could be construed as a potential conflict of interest.
